# Sociodemographic predictors of mental health service utilization among young adults with support in daily living in Sweden: a register-based study

**DOI:** 10.1186/s12888-025-07046-1

**Published:** 2025-06-05

**Authors:** Jenny Meyer, Sergej Engström, Maria Löthberg, Steve Berggren, Adriana Ramirez, Sonya Girdler, Tatja Hirvikoski, Sven Bölte, Ulf Jonsson

**Affiliations:** 1 Center of Neurodevelopmental Disorders (KIND), Centre for Psychiatry Research, Department of Women’s and Children’s Health, Karolinska Institutet & Stockholm Health Care Services, Region Stockholm, Stockholm, Sweden; 2 Department of Medical Sciences, Child and Adolescent Psychiatry, Uppsala University, Uppsala, Sweden; 3 Child and Adolescent Psychiatry, Stockholm Health Care Services, Region Stockholm, Stockholm, Sweden; 4 Curtin Autism Research Group, Curtin School of Allied Health, Curtin University, Perth, Australia; 5 Habilitation and Health, Region Stockholm, Stockholm, Sweden

**Keywords:** Neurodevelopmental, Emerging adulthood, Social services, Disability, Social determinants

## Abstract

**Background:**

An increasing number of young adults in Sweden are being granted support in daily living through social services. To enhance service planning and resource allocation for this growing group of young service users, we aimed to provide an overview of the social and health-related circumstances of young adults recently granted support in daily living. We had a particular focus on mental health service utilization and whether socio-demographic factors influence the use of care.

**Methods:**

Swedish nationwide register data were used to describe the sociodemographic characteristics and utilization of mental health services, including outpatient and inpatient care, treatment for suicidal behavior and pharmacological treatment. Sociodemographic predictors (sex, age, country of birth, education, and parental education) of mental health service utilization were explored using binary logistic regression.

**Results:**

All young adults (ages 18–29) granted support between 2017 and 2021 (*N* = 15,024; 49.2% female) were included. Unfavorable social circumstances were common, including truncated education, unemployment, and the need for financial assistance. Many had a history of psychiatric inpatient care (40.0%) and treatment for suicidal behavior (15.7%). The majority received psychiatric outpatient care (71.6%) and psychopharmacological treatment (73.9%) during their granting year. Common conditions for care included attention deficit hyperactivity disorder, anxiety and fear-related disorders, mood disorders, and autism spectrum disorder. Co-occurring conditions were prevalent. Utilization of mental health services was more common among females, particularly regarding treatment for suicidal behavior (adjusted OR 2.52; 95% CI 2.16–2.93). Higher educational level and being Swedish-born were associated with a greater likelihood of utilizing outpatient care and psychopharmacological treatment. In contrast, those born in Sweden were less likely than foreign-born to be admitted to inpatient care (adjusted OR 0.66; 95% CI 0.59–0.74).

**Conclusions:**

While social services recognize the support needs of this group of young adults, our findings suggest that sociodemographic characteristics such as sex, education level, and country of birth may facilitate or obstruct their access to mental health services. It is essential to coordinate efforts so that young adults with support in daily living can seek and access the mental health services they need.

**Clinical trial number:**

Not applicable.

**Supplementary Information:**

The online version contains supplementary material available at 10.1186/s12888-025-07046-1.

## Background

Emerging adulthood, typically occurring between ages 18 and 29 years, can be a period of increased opportunities and positive exploration [1]. However, young adults today confront high societal expectations in terms of skills proficiencies and academic requirements as they compete in job markets and strive towards independence [2–4]. In addition, many young adults experience mental, behavioral, and neurodevelopmental conditions that may affect their transition into adulthood [1, 2, 5–7]. Without effective support and treatment, these conditions are associated with several adverse psychosocial outcomes, including truncated education [8–10], labor market marginalization [8–11], economic burden [12, 13], poor adult health [8, 14], and death by suicide [15, 16]. In addition to the individual cost and suffering experienced by young people and their families, mental ill-health in young adults can result in societal costs and lost human potential [2, 13]. Hence, access to adequate mental health care and support is crucial.

In Sweden, the number of young adults being granted support in daily living has increased considerably over the past two decades [17]. Several possible explanations for this trend have been suggested, including a rise in mental health problems and related disabilities (or improved identification of these issues) [5, 17, 18], greater access to this form of support, and cuts to other services [17, 18]. Support in daily living, provided under the Swedish Social Services Act (2001:453) [19, 20], includes practical, pedagogical, and social support aiming to strengthen an individual’s abilities in managing everyday life at home and in the community [19]. This service targets individuals experiencing functional impairments, living in regular housing and is provided to males and females equally. While mental, behavioral, and neurodevelopmental conditions are common among service users, access to support does not depend on a specific diagnosis [17, 21]. Despite recognition that young adults are at a critical junction where adequate mental health care and support can influence their future opportunities and path in life [2], research on young service users is limited [22, 23], and their situation and support needs have not yet been thoroughly mapped.

Firstly, understanding the needs of this population requires a comprehensive description of their sociodemographic characteristics, including sex, country of birth, age, and education. Although the Swedish healthcare system is based on universal healthcare insurance emphasizing equal care based on needs [24, 25], there may still be barriers to accessing mental health services. Previous findings have indicated some potential sociodemographic determinants of mental health problems as well as mental health service utilization [5, 26–33]. However, there are some notable inconsistencies in the patterns of results. For example, mental health issues reportedly increase among young women, with females being more likely to utilize mental health services [5, 26–28]. Still, men are often overrepresented in the number of deaths by suicide [5, 29]. Despite some variation across studies, previous findings have indicated that foreign-born and ethnic minority groups may have an increased risk of severe mental illness and hospitalization, while they are underrepresented regarding utilization of outpatient psychiatric care and psychotropic medication [28, 30, 31]. Further, while lower socioeconomic status (including education) has been associated with an increased risk for mental health conditions [32], positive associations between higher education and mental health service utilization have been reported [26, 33]. Regarding age, recent findings have shown that Swedish young adults perceive more mental health issues and visit specialty psychiatric care to a greater extent than older adults. In contrast, psychopharmacological treatment, also provided within primary care, is more common among older adults [5]. Collectively, these findings highlight that less mental health service utilization may not always indicate better health and that predictors of mental health service utilization may vary across populations and with different types of care. Whether previous findings can be translated to young adults recently granted support in daily living is yet to be explored.

The overarching aim of our study was to provide a comprehensive overview of the social and health-related circumstances of young adults aged 18 to 29 who have recently been granted support in daily living. We especially focused on utilization of mental health services and the potential influence of sociodemographic factors. Our investigation had the following specific aims:

1. Provide a detailed description, including estimation of sex differences, regarding social circumstances, history of mental health care utilization, and access to mental health care during the granting year.
2. Explore sociodemographic predictors of mental health care utilization in this population.

## Methods

### Design

This study was based on Swedish nationwide register data. Sociodemographic characteristics and utilization of various mental health services were explored descriptively and compared by sex. Sociodemographic variables (sex, country of birth, age, and educational level of the young adults and their parents) were explored as potential predictors of mental health service utilization (outpatient care, inpatient care, treatment for suicidal behavior and pharmacological treatment) during the granting year. This study was reported in line with the Strengthening the Reporting of Observational Studies in Epidemiology (STROBE) guidelines [34].

### Study population

The study population comprised all young adults aged 18 to 29 years who: (a) were granted support in daily living between 2017 and 2021; (b) had not been granted support during the two preceding years; (c) were registered as residents in Sweden at the end of the granting year; and, (d) had a valid personal identity number or coordination number. We included a five-year period to increase the sample size and minimize the impact of temporary fluctuations.

### Setting

#### Support in daily living

In Sweden, social services within each municipality oversees and delivers this needs-based service. A social services care administrator assesses an individual’s eligibility for support and their needs based on interviews and medical statements. The support provided is tailored to meet individual needs, with the goal of promoting autonomy. The support typically entails guidance and practice in areas such as planning, establishing routines, managing household chores, and interacting with public authorities. It is available only to individuals living in regular housing (i.e., not individuals living in special housing), and the frequency of support varies between about once a month and several times a day. A support worker provides the support, primarily in the service user’s home [22, 23].

#### Mental health care in Sweden

In Sweden, mental health care is organized and delivered by each region via primary care and specialty out- or inpatient care. Although there is some overlap between these levels of care, individuals with mild to moderate mental health problems are usually treated within primary care, with those experiencing more severe and complex conditions supported through specialized care [25, 35]. Inpatient care is primarily intended for emergency situations, such as acute suicide risk, and includes both voluntary and compulsory admission.

#### Data sources

Data was obtained from Swedish nationwide registers held by the National Board of Health and Welfare [36] as well as Statistics Sweden [37]. The National Register of Care and Social Services for Elderly and Persons with Impairments was used to extract the study population and their age at inclusion. Information on sex and country of birth was retrieved from the Register of the Total Population. The Multi-Generation Register was used to link the population to children and parents. Data on the highest completed education (own and parents), employment, studies, and financial assistance were retrieved from the Longitudinal Integration Database for Health Insurance and Labor Market Studies. Data on psychiatric care was obtained from the National Patient Register, which includes inpatient care (nationwide since 1987) and visits to the doctor in specialty outpatient care since 2001 (including both private and public health care providers). Data on psychopharmacological treatment were retrieved from the Prescribed Drug Registry, covering drugs prescribed within primary- and specialty care.

## Measures

### Sociodemographic characteristics

The variables listed below were defined using data available for the year in which the support was granted (i.e., 2017–2021), except sex which was based on the latest available data: sex (male/female); the age in full years (continuous variable and dichotomized as ages 18 to 23 or 24 to 29); have own children (yes/no); country of birth (Swedish-born/foreign-born); the highest completed education (compulsory or less [up to 9 years], upper secondary [10–12 years], and tertiary [> 12 years]); being a student in the fall semester (yes/no); being employed (yes/no); receiving social assistance (yes/no); and activity compensation (yes/no).

Before 2018, which applies to all individuals in our population, compulsory education lasted nine years and began at the age of seven. Most students start upper secondary education around age 16. While upper secondary education is voluntary, it is often required for many jobs. Those finishing one to two years of upper secondary school and those completing a full upper secondary program of three years were included in the “upper secondary” category. Tertiary education encompasses all post-secondary education, including shorter vocational training as well as longer academic programs. In the regression analyses, the highest completed education of the service users was dichotomized (compulsory or less/upper secondary or more) due to the young age of some service users. Parental education was dichotomized as upper secondary or less/tertiary.

Social assistance and activity compensation are two distinct forms of financial assistance, which were reported separately and combined. Social assistance is granted by the social services and intended for households where basic living expenses (e.g., food, rent, medical care) cannot be covered by other means, whereas activity compensation is granted by the Social Insurance Office and intended for individuals between the ages of 19 to 29 with impaired work capacity. In addition, young people who have not yet completed their compulsory or upper secondary education due to a disability are entitled to activity compensation to complete their studies.

### Mental health service utilization

Utilization of psychiatric services was examined through broader indicators (i.e., psychiatric outpatient and inpatient care) alongside more specific and partly overlapping indicators (i.e., suicidal behavior, specific diagnoses, types of psychopharmacological treatment) to provide a more detailed description. The National Patient Register uses the International Classification of Diseases and Related Health Problems (ICD) to classify diagnoses and injuries associated with each care visit. As ICD-9 was used between 1987 and 1996 [38], data from this period were based on ICD-9 codes (i.e., only applies to the earliest data from before the granting year). All data on care visits that took place from 1997 onwards are based on ICD-10 codes [39], including all data from the granting year. In the result section, we used terminology per ICD-11 [40], when possible, to present the information in a way that is consistent with the current diagnostic system. However, the original ICD-9/ICD-10 codes are also provided for transparency.

#### Before the granting year

History of mental health service utilization was reported as occurring: (1) at any time prior to the granting year; (2) during adolescence (13–17 years of age); and (3) during childhood (< 13 years of age). Inpatient psychiatric care was defined as inpatient care that either took place within psychiatry or was coded with a primary diagnosis of a mental, behavioral, or neurodevelopmental condition (i.e., ICD-10: F00-F99/ICD-9: 290–319) or intentional self-harm (i.e., ICD-10: X60-X84/ICD-9: E950-E959). Treatment for suicidal behaviors was explored separately for intentional self-harm (out- and inpatient visits with an ICD code of ICD-10: X60-X84/ICD-9: E950-E959) and collapsed with care for injury with undetermined intent (out- and inpatient visits with an ICD code of ICD-10: Y10-Y34/ICD-9: E980-E989).

#### During the granting year

Specialty psychiatric care was defined as care visits that either took place within psychiatry or were coded with a main diagnosis of a mental, behavioral, or neurodevelopmental condition (ICD10: F00-F99) or intentional self-harm (ICD-10: X60-X84). This was explored separately for outpatient care and inpatient care, both as binary variables (yes/no) and continuous variables regarding the number of visits in outpatient care and the number of days remitted to inpatient care. Treatment for suicidal behaviors were binary variables, reported both separately for intentional self-harm (out- and inpatient visits with an ICD-10 code of X60-X84) and collapsed with Care for injury with undetermined intent (out- and inpatient visits with an ICD-10 code of Y10-Y34).

Care for specific mental, behavioral, and neurodevelopmental conditions was based on ICD-10 codes (F00-F99) registered in outpatient and inpatient care. Both primary and secondary diagnoses were included. Care for specific mental, behavioral, and neurodevelopmental conditions was presented as binary variables (yes/no), while the average number of diagnoses was continuous. Psychopharmacological treatment was based on dispensed prescribed drugs classified according to the Anatomical Therapeutic Chemical (ATC) classification system. The following ATC-codes were retrieved: Antipsychotics (N05A), Anxiolytics (N05B), Hypnotics and Sedatives (N05C), Antidepressants (N06A), Centrally acting sympathomimetics (N06BA), Drugs used for nicotine dependence (N07BA), Drugs used for alcohol dependence (N07BB), Drugs used for opioid dependence (N07BC). Psychopharmacological treatment was both binary (yes/no) and continuous (number of dispensed drugs from different drug groups).

### Data analyses

Descriptive statistics were used to present the distributions of sociodemographic data and different types of mental health service utilization in the population. For the categorical variables, sex differences were explored using the chi-square test. Due to some outliers and deviation from the normal distribution, the Mann-Whitney U test was used for the continuous variables.

Binary logistic regression was used to explore sex, age, country of birth, own education, and parental education as potential predictors of mental health service utilization during the granting year (outpatient care, inpatient care, treatment for suicidal behavior [X60-X84 or Y10-Y34], psychopharmacologic treatment). Both unadjusted and adjusted (including all the potential predictors) estimates (odds ratios; 95% confidence intervals) were calculated. An alpha level of *p* <.05 was used without adjustment for multiple comparisons.

### Sensitivity analyses

Missing data on the highest completed education was non-trivial (own: *n* = 1116, 7.4%; parental: *n* = 103, 0.7%). Known reasons for missing data on completed education include immigration to Sweden in adulthood, attending special needs schools, or being unable to complete any education due to illness or other reasons. We explored the impact of missing data in the binary logistic regression analyses by first excluding individuals with unknown education levels and thereafter included these individuals in the group categorized as having a lower level of education (i.e., “compulsory or less” regarding their own education and “upper secondary or less” for the parental education). As the results largely aligned, the latter was used in the main analyses to maintain the full sample.

## Results

### Study population

The study population included 15,024 individuals (49.2% females, mean age = 24 years). Since information on country of birth was missing for a few (*n* = 5), the comparative analyses regarding this variable and the regression analyses were based on 15,019 individuals.

### Sociodemographic characteristics

While data demonstrated some heterogeneity within the group (e.g., the full range of educational levels was represented), unfavorable socioeconomic circumstances were common: 41.9% lacked upper secondary education, 63.3% were neither working nor studying, and 70.7% had been granted financial assistance during their granting year. The social situation differed slightly between males and females, with a somewhat higher proportion of males having a low educational level, not working or studying, being foreign-born, and receiving social assistance. A higher proportion of the females had children (Table 1).

**Table 1:** Sociodemographic characteristics of the study population during the granting year

	Total sample (*N* = 15024)	Male (*n* = 7630)	Female (*n* = 7394)	Group comparison *p*-value
Mean age, SD	23.65 (3.31)	23.70 (3.30)	23.60 (3.31)	*0.065*
Age group, n (%)				*0.288*
Younger (18–23 years)	7261 (48.3)	3655 (47.9)	3606 (48.8)	
Older (24–29 years)	7763 (51.7)	3975 (52.1)	3788 (51.2)	
Country of birth, n (%)				*< 0.001*
Swedish-born	13,067 (87.0)	6459 (84.7)	6608 (89.4)	
Foreign-born	1952 (13.0)	1167 (15.3)	785 (10.6)	
Unknown^a^	5 (0.0)	4 (0.1)	1 (0.0)	
Have children, n (%)	1357 (9.0)	330 (4.3)	1027 (13.9)	*< 0.001*
Completed education, n (%)				*< 0.001*
Compulsory or less	6299(41.9)	3321 (43.5)	2978(40.3)	
Upper secondary	6278 (41.8)	3114 (40.8)	3164 (42.8)	
Tertiary	1331 (8.9)	562 (7.4)	769 (10.4)	
Unknown	1116 (7.4)	639 (8.3)	483 (6.5)	
Parental education, n (%)				*< 0.001*
Compulsory or less	1919 (12.8)	1139 (14.9)	780 (10.5)	
Upper secondary	6993 (46.5)	3441 (45.1)	3552 (48.0)	
Tertiary	6009 (40.0)	2997 (39.3)	3012 (40.7)	
Unknown	103 (0.7)	53 (0.7)	50 (0.7)	
Occupation, n (%)				
Employed	2683 (17.9)	1247 (16.3)	1436 (19.4)	*< 0.001*
Student	3281 (21.8)	1495 (19.6)	1786 (24.2)	*< 0.001*
One of the above	5515 (36.7)	2588 (33.9)	2927 (39.6)	*< 0.001*
Financial assistance, n (%)				
Social assistance	6047 (40.2)	3282 (43.0)	2765 (37.4)	*< 0.001*
Activity compensation	5826 (38.8)	2907 (38.1)	2919 (39.5)	*0.083*
One of the above	10,618 (70.7)	5548 (72.7)	5070 (68.6)	*< 0.001*

SD = Standard Deviation

^a^ Unknown country of birth is not included in the comparison between males and females

### Mental health service utilization

#### Before the granting year

A substantial proportion of the study population had been admitted to psychiatric inpatient care at some point before their granting year (40.0%) and/or had received care for suicidal behaviors (15.7%). Inpatient care and care for suicidal behaviors in young adulthood and adolescence were more prevalent among females (Table 2).

**Table 2:** History of mental health service utilization

Type of care	Total sample (*N* = 15024)	Male (*n* = 7630)	Female (*n* = 7394)	Group comparison *p*-value
Psychiatric inpatient care^a^, n (%)				
Before granting year	6012 (40.0)	2683 (35.2)	3329 (45.0)	< 0.001
During adolescence (13–17 years)	1871 (12.5)	602 (7.9)	1269 (17.2)	< 0.001
During childhood (< 13 years of age)	307 (2.0)	163 (2.1)	144 (1.9)	0.414
Treatment for intentional self-harm^b^, n (%)				
Before granting year	2066 (13.8)	618 (8.1)	1448 (19.6)	< 0.001
During adolescence (13–17 years)	793 (5.3)	159 (2.1)	634 (8.6)	< 0.001
During childhood (< 13 years of age)	136 (0.9)	27 (0.4)	109 (1.5)	< 0.001
Treatment for intentional self-harm or injury with undetermined intent^c^, n (%)				
Before granting year	2356 (15.7)	788 (10.3)	1568 (21.2)	< 0.001
During adolescence (13–17 years)	897 (6.0)	213 (2.8)	684 (9.3)	< 0.001
During childhood (< 13 years of age)	235 (1.6)	82 (1.1)	153 (2.1)	< 0.001

^a^ Psychiatric inpatient care = inpatient care that either took place within psychiatry or was coded with a primary diagnosis of a mental, behavioral or neurodevelopmental condition (i.e., ICD-10: F00-F99/ ICD-9: 290–319) or intentional self-harm (i.e., ICD-10: X60-X84/ ICD-9: E950-E959)

^b^ Treatment for intentional self-harm = out- and inpatient visits with an ICD code of ICD-10: X60-X84 / ICD-9: E950-E959

^c^ Treatment for injury with undetermined intent = out- and inpatient visits with an ICD code of ICD-10: Y10-Y34/ ICD-9: E980-E989

#### During the granting year

Most of the young adults received outpatient psychiatric care during the granting year (71.6%), and about one-fifth (20.5%) were admitted to inpatient psychiatric care. Both outpatient and inpatient care were significantly more common among women. Among those who received psychiatric care, males had slightly fewer care visits than females, while no significant sex difference was found regarding days of inpatient care. Care for suicidal behavior occurred in both sexes but was more than twice as common in females (7.9%) than in males (3.3%). A majority had received care for specific mental, behavioral, and neurodevelopmental conditions (71.2%), with the most prevalent conditions for care being attention deficit hyperactivity disorder (33.6%), anxiety and fear-related disorders (27.5%), mood disorders (27.3%), and autism spectrum disorder (23.7%). Co-occurring conditions were common. Females were overrepresented, except for a few conditions (e.g., psychotic disorders). A majority received psychopharmacological treatment; more than half were treated with antidepressants, more than a quarter with antipsychotics, and a majority received more than one type of psychotropic drug. A larger percentage of women received psychopharmacological treatment (80.1%) compared to men (67.9%) (Table 3).

**Table 3:** Mental health service utilization during the granting year

Type of care	Total sample (*N* = 15024)	Male (*n* = 7630)	Female (*n* = 7394)	Group comparison *p*-value
Psychiatric outpatient care^a^				
Visit in outpatient care, n (%)	10,753 (71.6)	5078 (66.6)	5675 (76.8)	*< 0.001*
Number of outpatient visits^b^, Median, (percentile 10, 90)	4 (10:1, 90:11)	3 (10:1, 90:10)	4 (10:1, 90:12)	*< 0.001*
Psychiatric inpatient care^a^				
Admission to inpatient care, n (%)	3087 (20.5)	1363 (17.9)	1724 (23.3)	*< 0.001*
Length of admission in days^c^, median, (percentile 10, 90)	19 (10:1, 90:104)	19 (10:1, 90:118)	18 (10:1, 90:100)	*0.596*
Treatment for suicidal behavior, n (%)^d^				
intentional self-harm	800 (5.3)	227 (3.0)	573 (7.7)	*< 0.001*
intentional self-harm or injury with undetermined intent	838 (5.6)	251 (3.3)	587 (7.9)	*< 0.001*
Specialty care for mental, behavioral and neurodevelopmental conditions^e^, n, (%)				
Visit for at least one condition (F00-F99)	10,700 (71.2)	5044 (66.1)	5656 (76.5)	*< 0.001*
Number of conditions from different categories^f^, Median, (percentile 10;90)	*2 (10:1*,* 90:4*)	*2 (10:1*,* 90:4*)	*2 (10:1*,* 90:4*)	*< 0.001*
Specialty care for specific conditions (ICD-10 code), n (%)				
Disorders due to substance use or addictive behaviors (F10-F19, F55)	1765 (11.7)	955 (12.5)	810 (11.0)	*0.003*
Schizophrenia or other primary psychotic disorders (F20-F29)	1204 (8.0)	822 (10.8)	382 (5.2)	*< 0.001*
Mood disorders (F30-F39)	4104 (27.3)	1744 (22.9)	2360 (31.9)	*< 0.001*
Anxiety and fear related disorders (F40-F41, F93)	4126 (27.5)	1473 (19.3)	2653 (35.9)	*< 0.001*
Obsessive compulsive disorder (F42)	657 (4.4)	276 (3.6)	381 (5.2)	*< 0.001*
Reaction to severe stress, and adjustment disorders (F43)	1763 (11.7)	482 (6.3)	1281 (17.3)	*< 0.001*
Feeding or eating disorders (F50)	478 (3.2)	40 (0.5)	438 (5.9)	*< 0.001*
Sleep-wake disorders (F51)	208 (1.4)	100 (1.3)	108 (1.5)	*0.431*
Personality disorders and related traits (F60-F62, F69)	1332 (8.9)	219 (2.9)	1113 (15.1)	*< 0.001*
Gender identity disorders (F64)	279 (1.9)	140 (1.8)	139(1.9)	*0.838*
Disorders of intellectual development (F70-F79)	827 (5.5)	422 (5.5)	405 (5.5)	*0.886*
Autism spectrum disorder (F84.0, F84.1, F84.5)	3562 (23.7)	1829 (24.0)	1733 (23.4)	*0.442*
Attention deficit hyperactivity disorder (F90, F98.8)	5054 (33.6)	2395 (31.4)	2659 (36.0)	*< 0.001*
Other Neurodevelopmental disorders (F80-F83, F843, F844, F848, F849, F88-F89)	495 (3.3)	263 (3.4)	232 (3.1)	*0.288*
Mental, behavioral or neurodevelopmental disorders, unspecified (F99-F99)	258 (1.7)	125 (1.6)	133 (1.8)	*0.449*
Conditions occurring in less than 1% (F00-F09, F44, F45, F48, F52, F63, F65, F66, F68, F90-F92, F94, F95, F98)	688 (4.6)	313 (4.1)	375 (5.1)	*0.004*
Dispensed prescribed drugs				
Psychopharmacologic treatment^g^, n (%)	11,103 (73.9)	5182 (67.9)	5921 (80.1)	*< 0.001*
Number of drugs from different drug groups^h^, Median, (percentile 10;90)	2 (10:1, 90:4)	2 (10:1, 90:4)	2 (10:1, 90:4)	*< 0.001*
Specific dispensed prescribed drugs (ATC-code), n (%)				
Antipsychotics (N05A)	4099 (27.3)	1930 (25.3)	2169 (29.3)	*< 0.001*
Anxiolytics (N05B)	3415 (22.7)	1252 (16.4)	2163 (29.3)	*< 0.001*
Hypnotics and Sedatives (N05C)	5646 (37.6)	2388 (31.3)	3258 (44.1)	*< 0.001*
Antidepressants (N06A)	7773 (51.7)	3325 (43.6)	4448 (60.2)	*< 0.001*
Centrally acting sympathomimetics (N06BA)	3778 (25.1)	1745 (22.9)	2033 (27.5)	*< 0.001*
Drugs used for nicotine dependence (N07BA)	57 (0.4)	22 (0.3)	35 (0.5)	*0.065*
Drugs used for alcohol dependence (N07BB)	242 (1.6)	115 (1.5)	127 (1.7)	*0.306*
Drugs used for opioid dependence (N07BC)	51 (0.3)	30 (0.4)	21 (0.3)	*0.250*

^a^ Psychiatric care = visits in specialty care that took place within the psychiatric field of activity and/or were coded with a main diagnosis of ICD-10: F00-F99

or with ICD-10 code of X60-X84

^b^ Number of outpatient visits is based on those who received outpatient care

^c^ Length of admission is based on those who were admitted to inpatient care

^d^ Treatment for suicidal behaviors include out- and inpatient visits with an ICD-10 code of X60-X84 or Y10-Y34

^e^ Specialty care for mental, behavioral, and neurodevelopmental conditions includes out- and inpatient care visits coded with a main and/or secondary diagnosis of ICD-10:F00-F99

^f^ Number of diagnoses from different categories is based on those with at least one recorded diagnosis and refers to the diagnostic categories presented in the table

^g^ Psychopharmacologic treatment = dispensed drugs with any of the following ATC-codes (N05A-N05C, N606A, N06BA, N07BA-N07C)

^h^ Number of drugs from different drug groups is based on those who had at least one prescribed drug and refers to the drug groups presented in the table

### Predictors of mental health service utilization

Results from the unadjusted and adjusted regression models are presented in Table 4, and the corresponding sensitivity analyses in Additional file 1. Even after adjusting for country of birth, age, own education, and parental education, being female was positively associated with all explored types of mental health service utilization, particularly regarding care for suicidal behavior (aOR 2.52; 2.16–2.93). Care for suicidal behaviors was less common in the older age group (aOR 0.71; 0.62–0.82), whereas psychopharmacological treatment was predicted by older age (aOR 1.26; 1.16–1.36). Being Swedish-born predicted utilization of outpatient care (aOR 1.30; 1.17–1.44) and pharmacological treatment (aOR 1.48; 1.34–1.64), while inpatient care was more common among the foreign-born (aOR 0.66; 0.59–0.74). Moreover, higher educational level (both own and parental) was associated with a higher likelihood of out- and inpatient psychiatric care as well as pharmacological treatment, with particularly strong associations for the latter (own education: aOR 1.52; 1.40–1.64, parental education: aOR 1.44; 1.33–1.56). However, the association between own education and inpatient care was not confirmed by the sensitivity analyses conducted without those with unknown education (Additional file 1).

**Table 4:** Predictors of mental health service utilization (*n* = 15019)

	Outpatient care^a^ OR (95 CI), *p*-value	Inpatient care^b^ OR (95 CI), *p*-value	Treatment for suicidal behavior^c^ OR (95 CI), *p*-value	Psychopharmacological treatment^d^ OR (95 CI), *p*-value
Sex (reference: male)				
Unadjusted model	1.66 (1.55–1.78), *< 0.001*	1.40 (1.29–1.52), *< 0.001*	2.53 (2.18–2.95), *< 0.001*	1.90 (1.76–2.05), *< 0.001*
Adjusted model^e^	1.62 (1.50–1.74), *< 0.001*	1.42 (1.31–1.54), *< 0.001*	2.52 (2.16–2.93), *< 0.001*	1.85 (1.71–1.99), *< 0.001*
Age group (reference: younger)				
Unadjusted model	1.08 (1.00-1.15), *0.045*	1.08 (0.99–1.17),.*054*	0.72 (0.63-0.083), *< 0.001*	1.40 (1.30–1.51), *< 0.001*
Adjusted model^e^	0.98 (0.91–1.06),.*641*	1.06 (0.98–1.16), *0.140*	0.71 (0.62–0.82), *< 0.001*	1.26 (1.16–1.36), *< 0.001*
Country of birth (reference: foreign-born)				
Unadjusted model	1.47 (1.33–1.62), *< 0.001*	0.72 (0.64–0.80), *< 0.001*	1.08 (0.88–1.34), *0.465*	1.77 (1.60–1.96), *< 0.001*
Adjusted model^e^	1.30 (1.17–1.44), *< 0.001*	0.66 (0.59–0.74), *< 0.001*	0.99 (0.80–1.23), *0.952*	1.48 (1.34–1.64), *< 0.001*
Own education (reference: lower education)				
Unadjusted model	1.49 (1.39–1.60), *< 0.001*	1.18 (1.09–1.28), *< 0.001*	1.04 (0.91–1.20), *0.545*	1.78 (1.65–1.91), *< 0.001*
Adjusted model^e^	1.39 (1.29–1.50), *< 0.001*	1.15 (1.06–1.25), *0.001*	1.08 (0.93–1.25), *0.318*	1.52 (1.40–1.64), *< 0.001*
Parental education (reference: lower education)				
Unadjusted model	1.34 (1.25–1.44), *< 0.001*	1.19 (1.10–1.29), *< 0.001*	1.07 (0.93–1.23), *0.356*	1.59 (1.47–1.71), *< 0.001*
Adjusted model^e^	1.24 (1.15–1.34), *< 0.001*	1.19 (1.09–1.29), *< 0.001*	1.05 (0.91–1.21), *0.535*	1.44 (1.33–1.56), *< 0.001*

CI = Confidence Interval; OR = Odds Ratio

^a^ Outpatient visits that took place within psychiatric field of activity and/or were coded with a main diagnosis of ICD-10: F00-F99 or with an ICD-10 code of X60-X84

^b^ Inpatient visits that took place within psychiatric field of activity and/or were coded with a main diagnosis of ICD-10: F00-F99 or with an ICD-10 code of X60-X84

^c^ Treatment for suicidal behaviors include out- and inpatient visits with an ICD-10 code of X60-X84 or Y10-Y34

^d^ Psychopharmacologic treatment refers to dispensed drugs with any of the following ATC-codes: N05A-N05C, N606A, N06BA, and N07BA-N07C

^e^ Adjusted models include sex, age, country of birth, own education and parental education

## Discussion

This is the first register-based study providing a detailed description of the social and health-related circumstances of young adults (ages 18–29) with support in daily living. Combining data from several nationwide registers, including the entire population of interest, we aimed to describe their situation when entering the support service. Our findings suggest that they face various unfavorable social circumstances, such as a low level of education, unemployment, and economic hardships. Further, a substantial proportion had a history of psychiatric inpatient care and treatment of suicidal behavior, for some already at an early age. Mental health service utilization during the granting year was common. Being female and having a higher educational level was associated with an increased likelihood of receiving mental health care, while associations with country of birth and age varied depending on the form of care.

Recent years have seen a reported increase in mental health problems among young adults in Sweden and globally [2, 5]. National data from 2022 of Swedish young adults (ages 18–29) show that about 8% of the women and 5.5% of the men used specialty psychiatric care, while about 13% of the women and 6% of the men were treated with antidepressants and about 0.34% of the women and 0.16% of the men had received care for intentional self-harm [5]. These statistics emphasize the need for societal investments in promoting functioning and mental health in young individuals in general [2, 35, 41]. When contrasted to our findings, they also illustrate the particularly vulnerable situation of young service users with support in daily living. Given the identified support needs of the study population, our results showing high mental health care utilization were partly expected. Still, the full extent of mental health service use was previously unknown, revealing the early onset of severe mental health problems in this group. In addition, our results indicated complex needs. For instance, a significant proportion neither worked nor studied (63.3%), compared to about 7.7–8.4% of all young adults in Sweden (aged 20–29) in 2024 [42] and a relatively large proportion (41.9%) lacked upper secondary education. Our findings emphasize the importance of early detection and treatment of mental ill-health, as well as interventions that address support needs in education, workforce entry, and the overall transition to adulthood [2, 11, 41, 43, 44].

Furthermore, our findings suggest some potential social determinants of mental health service utilization within this group of service users. Even though daily living support is equally common among young men and women, female service users were more likely to utilize all explored types of care. Our results are in line with previous reports showing that young women generally report more mental health problems and seek more care [5, 26–28]. However, it has been suggested that men may underuse mental health care [26, 28], especially given their overrepresentation in suicide rates [5, 29]. Hence, potential barriers to mental health care use for males with support in daily living must be considered, such as gender norms in help-seeking behaviors [28], limited trust in mental health services [26], and lack of mental health literacy [26, 28]. In addition, gender biases in clinical practice (e.g., diagnostic procedure and treatment) have been indicated by previous research [45], which requires further investigation. Further, higher educational attainment among the service users and their parents was positively associated with the utilization of specialty psychiatric care and psychopharmacological treatment. Given these results as well as previous findings [26, 33], it is reasonable to believe that education may facilitate access to care by fostering parental advocacy, improving the ability to identify and communicate needs and navigate the healthcare system and increasing knowledge about one’s rights. Subgroups at risk of underusing mental health care may benefit from interventions aiming at increasing mental health literacy (including identifying symptoms and when and where to seek care) [28, 46, 47].

Some of the associations were less clear-cut. For example, foreign-born individuals were overrepresented in inpatient care, while Swedish-born individuals utilized more outpatient care and pharmacological treatment. Barriers for foreign-born individuals to accessing care may lead some to seek or receive care only when this is inevitable [30]. Cultural factors (e.g., views on mental health, stigma, and preferences for support) [26, 48, 49], structural barriers (e.g., costs) [26], familiarity with the country’s health care system [31, 46], bias and discrimination within the health care system [50], and the service’s readiness to respond to specific needs based on ethnic background (e.g., access to qualified interpreters, cultural competency) [46, 51] are all factors that need further investigation. A Swedish longitudinal study [31] found that time in the country and region of origin affected mental health service utilization, which was not accounted for in the present study. In line with previous findings, our study suggested that care for suicidal behaviors was more common in the younger age group as well as among females [5]. Conversely, the likelihood of psychopharmacological treatment increased with age, consistent with previous reports [5]. This pattern may result from stepped care, wherein older individuals have had more time to explore treatment options [52]. Additionally, given that our data refer to dispensed drugs, our findings may also reflect lower compliance with psychotropic medications among younger individuals [53].

The sociodemographic determinants of mental health care utilization studied here show a pattern of results consistent with previous findings in the general population. It is unclear, however, why this pattern should persist in this highly selected population with identified support needs. This may suggest that there are systematic barriers to accessing mental health services in Sweden. The complex social and mental health-related needs indicated by our findings will, in many cases, require parallel support from multiple service providers (e.g., social support, supported education and employment, as well as psychological and pharmacological treatment), where effective coordination of services will be crucial in achieving desired outcomes [22, 50]. In such coordination, the support worker (i.e., providing the daily living support) can be a key player, often meeting the service users in their home environment weekly (sometimes daily). Support workers are well placed to both identify the early signs of deteriorating mental health (e.g., impaired functioning and suicidal ideation) and facilitate contact with psychiatry when needed [23]. For this to succeed, support workers must receive adequate training and supervision, service users must be willing to accept such support, and mental health services should be available when needed.

### Limitations

The results should be interpreted in light of some limitations. First, the lack of a control group prevents us from estimating differences between the target and the general population, although official statistics enable rough comparisons [5, 42]. Second, the indicators studied here do not cover the full range of mental health care utilization. The Swedish National Patient Register only covered visits to medical doctors in specialty care, omitting other care contacts (e.g., psychologists and occupational therapists). Similarly, primary care, which administers about 70% of the treatments of depression and anxiety disorders in adults [52], was only covered through prescribed medication. In addition, we do not have information about the quality of the care provided. Third, while register data can broadly describe the population of interest, it lacks in-depth information on the participants’ experiences and perceived needs, including perceived barriers to accessing care. Qualitative studies and surveys are warranted as a complement to the current study to better understand the complex mental health needs of young service users. Fourth, barriers to mental health care utilization extend beyond the factors examined in this study [50, 54]. For example, structural accessibility factors such as the density of care providers, opening hours, and travel time can affect care utilization [54]. Additionally, barriers at different levels may interact, warranting further research [50, 54]. Fifth, while a longitudinal design was beyond the scope of this study, future research is needed to investigate long-term psychosocial outcomes in young service users. Finally, it is uncertain whether this pattern of results applies to other healthcare systems and cultural contexts. However, young individuals in many countries face similar challenges related to mental health in transitioning to adulthood [2].

## Conclusions

Overall, this study shows that young adults with recently granted support in daily living are a vulnerable group facing several socioeconomic and mental health-related challenges. While these findings imply a need for multimodal support and treatment, both prevention and rehabilitation, the perceived support needs among young service users should be explored further. Moreover, our findings suggest that sociodemographic factors such as sex, education, and country of birth can facilitate or hinder access to mental health care for these young adults. Potential barriers to mental health service utilization among young service users need to be addressed in both social services and mental health services to ensure that those in need of care are adequately supported to seek and access treatment.

## Electronic supplementary material

Below is the link to the electronic supplementary material.

Supplementary Material 1

## Data Availability

National regulations regarding data retrieved from the Swedish registries prevent us from sharing any data openly, due to reasons related to confidentiality and protection of human privacy.

## Abbreviations

aOR: Adjusted Odds ratio
ATC: Anatomical Therapeutic Chemical
CI: Confidence Interval
ICD: International Classification of Diseases and Related Health Problems
OR: Odds ratio
SD: Standard deviation
