# Supplementary material for: Sociodemographic predictors of mental health service utilization among young adults with support in daily living in Sweden: a register-based study
Source: BMC Psychiatry. 2025 Jun 5;25:580. doi: 10.1186/s12888-025-07046-1 (PMC12139090; doi:10.1186/s12888-025-07046-1)
Supplement: Supplementary file 1 — Supplementary Material 1 [file 12888_2025_7046_MOESM1_ESM.docx]

|  | **Outpatient care^a^**  OR (95 CI), *p-value* | **Inpatient care^b^**  OR (95 CI), *p-value* | **Treatment for suicidal behavior^c^**  OR (95 CI), *p-value* | **Psychopharmacological treatment^d^**  OR (95 CI), *p-value* |
| --- | --- | --- | --- | --- |
| Sex (reference: male) |  |  |  |  |
| Unadjusted model | 1.71 (1.58-1.84), *<.001* | 1.40 (1.29-1.52), *<.001* | 2.49 (2.13-2.90), *<.001* | 2.01 (1.86-2.18), *<.001* |
| Adjusted model^e^ | 1.68 (1.55-1.81), *<.001* | 1.42 (1.31-1.54), *<.001* | 2.48 (2.12-2.90), *<.001* | 1.97 (1.82-2.14), *<.001* |
| Age group (reference: younger) |  |  |  |  |
| Unadjusted model | 1.05 (0.97-1.13), *.219* | 1.09 (1.00-1.18), .*045* | 0.72 (0.63-0.83), *<.001* | 1.43 (1.32-1.54), *<.001* |
| Adjusted model^e^ | 1.02 (0.94-1.10), .*623* | 1.10 (1.01-1.19), *.037* | 0.73 (0.63-0.85), *<.001* | 1.36 (1.25-1.47), *<.001* |
| Country of birth (reference: foreign-born) |  |  |  |  |
| Unadjusted model | 1.44 (1.29-1.61), *<.001* | 0.71 (0.63-0.80), *<.001* | 1.08 (0.86-1.36), *.495* | 1.72 (1.54-1.92), *<.001* |
| Adjusted model^e^ | 1.32 (1.17-1.48), *<.001* | 0.67 (0.59-0.75), *<.001* | 1.00 (0.79-1.26), *.990* | 1.50 (1.33-1.68), *<.001* |
| Own education (reference: lower education) |  |  |  |  |
| Unadjusted model | 1.20 (1.11-1.30), *<.001* | 1.07 (0.99-1.16), *.094* | 0.95 (0.82-1.09), *.465* | 1.49 (1.37-1.61), *<.001* |
| Adjusted model^e^ | 1.11 (1.03-1.21), *.008* | *1.03 (0.95-1.12), .490* | 0.98 (0.84-1.14), *.775* | 1.26 (1.16-1.37), *<.001* |
| Parental education (reference: lower education) |  |  |  |  |
| Unadjusted model | 1.31 (1.21-1.41), *<.001* | 1.17 (1.08-1.27), *<.001* | 1.04 (0.90-1.21), *.557* | 1.52 (1.41-1.65), *<.001* |
| Adjusted model^e^ | 1.26 (1.16-1.37), *<.001* | 1.18 (1.09-1.29), *<.001* | 1.03 (0.89-1.20), *.652* | 1.44 (1.32-1.57), *<.001* |

**Supplementary Table 1** Predictors of mental health service utilization analyzed without individuals with unknown education level (n =13820)

CI = Confidence Interval; OR= Odds Ratio

^a^ Outpatient visits that took place within the psychiatric field of activity and/or were coded with a main diagnosis of ICD-10: F00-F99 or with an ICD-10 code of X60-X84

^b^ Inpatient visits that took place within psychiatric field of activity and/or were coded with a main diagnosis of ICD-10: F00-F99 or with an ICD-10 code of X60-X84

^c^ Treatment for suicidal behaviors include out- and inpatient visits with an ICD-10 code of X60-X84 or Y10-Y34

^d^ Psychopharmacologic treatment refers to dispensed drugs with any of the following ATC-codes: N05A-N05C, N606A, N06BA, and N07BA-N07C

^e^ Adjusted models include sex, age, country of birth, own education and parental education
